# Supplementary material for: Quantifying audio visual alcohol imagery in popular Indian films: a content analysis
Source: BMJ Open. 2021 Jun 2;11(5):e040630. doi: 10.1136/bmjopen-2020-040630 (PMC8183268; doi:10.1136/bmjopen-2020-040630)
Supplement: Supplementary data [file bmjopen-2020-040630supp001.pdf]

Characteristics of the Coded Films for Alcohol Imagery

Film Name	Language	Genre	Age Rating	Total number of 5 minutes intervals	Number and Percentage of Alcohol Imagery
Ae Dil Hai Mushkil	Hindi	Drama, Musical Music	UA	31	24(77.4)
Raees	Hindi	Action, Crime, Drama	UA	27	17(63.0)
Kabali	Tamil	Drama, Crime	UA	29	16(55.2)
Tanu Weds Manu	Hindi	Drama, Comedy, Romance	UA	25	13(52.0)
Rustom	Hindi	Crime, Drama, Thriller	UA	29	13(44.8)
Welcome Back	Hindi	Drama, Crime	UA	29	10(34.5)
Pulimurugan	Malyalam	Action	U	33	10(30.3)
Juduwaa 2	Hindi	Action, Comedy	UA	30	9(30.0)
Shrimanthudu	Telugu	Drama, Action	UA	32	9(28.1)
Airlift	Hindi	Drama, History	UA	25	6(24.0)
Kabil	Hindi	Drama	UA	27	6(22.2)
Prem Ratan Dan Payo	Hindi	Action, Drama, Musical	U	32	6(18.8)
Mersal	Tamil	Action, Thriller	U	34	6(17.6)
Shivaay	Hindi	Action, Adventure, Drama	UA	30	5(16.7)
Bahubali	Telugu	Action, Drama, Fantasy	UA	31	5(16.1)
Dilwale	Hindi	Action, Comedy	UA	32	5(15.6)
Dangal	Hindi	Action, Biography, Drama	U	33	5(15.2)
Tubelight	Hindi	Drama, History	U	27	4(14.8)
Baby	Hindi	Action, Thriller	UA	32	4(12.5)
Sulthan	Hindi	Drama, Sport	UA	33	4(12.1)
I	Tamil	Romantic Thriller	U	34	4(11.8)
Their	Tamil	Action	UA	31	3(9.7)
M S Dhoni	Hindi	Biography, Drama, Sport	U	36	3(8.3)
Bajirao Mastani	Hindi	Drama, Comedy, Action	UA	32	2(6.3)
Bahubali 2	Telugu	Action, Drama, Fantasy	UA	34	2(5.9)
Toilet Ek Pream Katha	Hindi	Comedy, Drama	UA	30	1(3.3)
Goolmaal Again	Hindi	Comedy	UA	31	1(3.2)
Bhajarangi Bhaijan	Hindi	Drama, Comedy, Action	UA	31	1(3.2)
Tiger Zinda Hai	Hindi	Action, Thriller	UA	33	1(3.0)
Secret Super Star	Hindi	Drama	UA	30	0(0.0)
