# Supplementary material for: Quantifying audio visual alcohol imagery in popular Indian films: a content analysis
Source: BMJ Open. 2021 Jun 2;11(5):e040630. doi: 10.1136/bmjopen-2020-040630 (PMC8183268; doi:10.1136/bmjopen-2020-040630)
Supplement: Author's manuscript [file bmjopen-2020-040630.draft_revisions.pdf]

BMJ Open

BMJ Open is committed to open peer review. As part of this commitment we make the peer review history of every article we publish publicly available.

When an article is published we post the peer reviewers' comments and the authors' responses online. We also post the versions of the paper that were used during peer review. These are the versions that the peer review comments apply to.

The versions of the paper that follow are the versions that were submitted during the peer review process. They are not the versions of record or the final published versions. They should not be cited or distributed as the published version of this manuscript.

BMJ Open is an open access journal and the full, final, typeset and author-corrected version of record of the manuscript is available on our site with no access controls, subscription charges or pay-per-view fees (http://bmjopen.bmj.com).

If you have any questions on BMJ Open's open peer review process please email info.bmjopen@bmj.com

BMJ Open

Audio Visual Alcohol Content in Popular Indian Films

Journal:	*BMJ Open*
Manuscript ID	bmjopen-2020-040630
Article Type:	Original research
Date Submitted by the Author:	29-May-2020
Complete List of Authors:	Bhagawath, Rohith; Kasturba Medical College Manipal, Community Medicine<br>Kulkarni, Muralidhar; Kasturba Medical College Manipal, Community Medicie<br>Britton, John; UK Center for Tobacco and Alcohol Studies, Division of Epidemiology and Public Health ; University of Nottingham<br>Cranwell, Jo; University of Bath, Department for Health<br>Arora, Monika; HRIDAY, ; Public Health Foundation of India, Health Promotion<br>Nazar, Gaurang; HRIDAY<br>Mullapudi, Somya; Kasturba Medical College Manipal, Community Medicine<br>Kamath, Veena; Kasturba Medical College Manipal, Community Medicine
Keywords:	Health policy < HEALTH SERVICES ADMINISTRATION & MANAGEMENT, EPIDEMIOLOGY, PUBLIC HEALTH, Child & adolescent psychiatry < PSYCHIATRY, Community child health < PAEDIATRICS

SCHOLARONE™
Manuscripts

I, the Submitting Author has the right to grant and does grant on behalf of all authors of the Work (as defined in the below author licence), an exclusive licence and/or a non-exclusive licence for contributions from authors who are: i) UK Crown employees; ii) where BMJ has agreed a CC-BY licence shall apply, and/or iii) in accordance with the terms applicable for US Federal Government officers or employees acting as part of their official duties; on a worldwide, perpetual, irrevocable, royalty-free basis to BMJ Publishing Group Ltd ("BMJ") its licensees and where the relevant Journal is co-owned by BMJ to the co-owners of the Journal, to publish the Work in this journal and any other BMJ products and to exploit all rights, as set out in our licence.

The Submitting Author accepts and understands that any supply made under these terms is made by BMJ to the Submitting Author unless you are acting as an employee on behalf of your employer or a postgraduate student of an affiliated institution which is paying any applicable article publishing charge ("APC") for Open Access articles. Where the Submitting Author wishes to make the Work available on an Open Access basis (and intends to pay the relevant APC), the terms of reuse of such Open Access shall be governed by a Creative Commons licence – details of these licences and which Creative Commons licence will apply to this Work are set out in our licence referred to above.

Other than as permitted in any relevant BMJ Author's Self Archiving Policies, I confirm this Work has not been accepted for publication elsewhere, is not being considered for publication elsewhere and does not duplicate material already published. I confirm all authors consent to publication of this Work and authorise the granting of this licence.

For peer review only

Audio Visual Alcohol Content in Popular Indian Films

Rohith Bhagawath[1], Muralidhar Kulkarni[1], John Britton[2*], Jo Cranwell[3], Monika Arora[4,5], Gaurang P Nazar[4], Somya Mullapudi[1], Veena G Kamath[1].

Correspondence to:

Professor John Britton

UK Center for Tobacco and Alcohol Studies, Division of Epidemiology and Public Health,

University of Nottingham

E-mail: j.britton@outlook.com

[1] Department of Community Medicine, Kasturba Medical College, MAHE, Manipal, India

[2] Division of Epidemiology and Public Health, University of Nottingham

For peer review only

[3] Tobacco Control Research Group, Department for Health, University of Bath, Bath, UK

[4] HRIDAY, Delhi, India

[5] Health Promotion and Tobacco Control, Public Health Foundation of India, Delhi, India

For peer review only

For peer review only - http://bmjopen.bmj.com/site/about/guidelines.xhtml

Word Count: 1804

Declaration of Interests/Competing Interest Statement: None to declare.

Key words: Alcohol, Coding, Films, Adolescents, Media, Imagery.

Funding Statement:

This work was supported by the Medical Research Council [grant number MR/P008933/1] of the United Kingdom under the Global Alliance for Chronic Lung diseases program.

Contributors: JB, MMK and RB conceptualised the study. The coding sheet was developed by RB, MMK, JC and GPN. Coding analysis was done by RB, MMK and VGK. Interpretation of data and drafting the manuscript was done by RB, MMK, VGK, SM and MA. Final approval of the version was read and approved by all authors.

Data Sharing Statement: Additional data on the alcohol content found in each film are available on request from rohit.bhagawath@manipal.edu.

For peer review only - http://bmjopen.bmj.com/site/about/guidelines.xhtml

For peer review only

For peer review only - http://bmjopen.bmj.com/site/about/guidelines.xhtml

Abstract

Objectives: Though exposure to alcohol imagery in films is a significant determinant of uptake and severity of alcohol consumption among young people, but there is poor evidence regarding the content of alcohol imagery in films in low and middle-income countries. We have measured alcohol imagery content and branding in popular Indian films, in total and in relation to language and age rating.

Design: In this observational study we measured alcohol imagery semi-quantitatively using 5-minute interval coding. We coded each interval according to whether it contained alcohol imagery or brand appearances.

Setting: India.

Participants: None. Content analysis of a total of 30 National Box office hit films over a period of three years from 2015-17.

Primary and secondary outcome measures: Quantum of alcohol imagery in Indian films and its distribution in relation to age and language rating has been determined.

Results: The 30 films included 22 (73%) Hindi films and eight (27%) in regional languages. Seven (23%) were rated suitable for viewing by all ages (U), and 23 (77%)

For peer review only - http://bmjopen.bmj.com/site/about/guidelines.xhtml

rated as suitable for viewing by children subject to parental guidance for those aged under 12 (UA). Any alcohol imagery was seen in 97% of the films, with 195 of a total of 923 5-minute intervals, and actual alcohol use in 25 (83%) films, in 90 (10%) intervals. The occurrence of these and other categories of alcohol imagery was similar in U and UA-rated films, and in Hindi and local language films. Episodes of alcohol branding occurred in ten intervals in five films.

Conclusion: Almost all films popular in India contain alcohol imagery, irrespective of age rating and language. Measures need to be undertaken to limit alcohol imagery in Indian films to protect the health of young people, and to monitor alcohol imagery in other social media platforms in future.

Strengths and limitations of this study:

- Our study, to the best of our knowledge is the first of its kind to measure alcohol imagery in popular films in India.
- The study demonstrates that almost all popular Indian films contain alcohol imagery.
- We included only top ten national box office hit films in the years 2015, 2017 and 2017 since coding films is time-consuming.

For peer review only - http://bmjopen.bmj.com/site/about/guidelines.xhtml

For peer review only

- Although our study did not include any A (Adult) or S-rated (Restricted to specialised audience) films, our findings related entirely to films classified by the CBFC as suitable for young people.

For peer review only

For peer review only - http://bmjopen.bmj.com/site/about/guidelines.xhtml

Background:

Consumption of alcohol is a major avoidable cause of morbidity and mortality [1]. According to the WHO, there are 2.3 billion current alcohol users around the world [2], and in 2016 alcohol consumption led to 2.8 million deaths [3]. Global alcohol consumption has increased by 38% in the last decade [4], indicating that the burden of morbidity and mortality caused by alcohol is likely to grow.

Alcohol consumption is a significant public health problem in India [5]. Although rare among women, nearly one in three men now consume alcohol [6], with an average intake of 18.3 litres per year[2]. Although the mean age at which people begin consuming alcohol is 21 years[7,8], 1.3% of children aged 10-17 report alcohol consumption [9], and studies reveal that early onset of use of alcohol in India correlates with chronic heavy drinking patterns later in adult life[7,8].

Research among adolescents in developed countries indicates that in addition to paid-for advertising, exposure to media imagery of alcohol products and alcohol consumption, particularly in films, plays an important role in promoting the uptake of alcohol consumption among young people[10]. However, while national film age-rating or classification systems typically include restrictions on alcohol imagery allowed in

For peer review only - http://bmjopen.bmj.com/site/about/guidelines.xhtml

films rated suitable for viewing by children or young people, analysis of alcohol imagery in popular UK films demonstrates that alcohol content continues to occur frequently in films marketed to these groups[11]. To our knowledge, however there here has to date been no evaluation of the extent to which popular films in India include alcohol imagery. This study was therefore carried out to quantify alcohol imagery, in relation to the age classification and language of the film, in a sample of the most popular films in India in the years 2015-2017.

Methods:

After obtaining Institutional ethics approval (IEC : 893/2018), we used Indian national box office ratings data to identify the top 10 Indian films by box office revenue in each of the years 2015, 2016 and 2017 [12]. The CBFC (Central Board of Film Certification) age rating (comprising U (Unrestricted Public Exhibition), UA (Parental Guidance for children below the age of 12 years), A (Restricted to adults) and S (Restricted to any special class of persons)) along with the genre and language of each film was noted. We used 5-minute interval coding, as described in previous studies[13,14], to code the presence of alcohol imagery in each interval as any use, actual use, implied use, other

For peer review only

For peer review only - http://bmjopen.bmj.com/site/about/guidelines.xhtml

alcohol references and alcohol brand appearances. Actual use of alcohol was coded if an actor/actress in the film was shown consuming alcohol, while implied alcohol use typically involved verbal comments related to alcohol or non-verbal actions such as holding a glass or a bottle appearing to contain an alcoholic drink. Other alcohol references usually comprised the appearance of alcohol bottles or beer mugs. An occurrence in any of the above categories was coded as present or absent for each interval. Multiple appearances in the same category in the same interval were coded as a single event, and appearances in different categories as separate events. The same appearance transitioning into two or more intervals was coded as two or more events, as appropriate. For alcohol branding we noted occurrences of clear, unambiguous alcohol branding on-screen, sub-grouped as branding on a product used in a scene, branding on a product not used in a scene, branded merchandise, advertisements visible in scenes of alcohol content and any other alcohol-related advertisement. The total number of alcohol brands shown in were counted and listed.

Patient and public involvement: No Patients were involved in this study.

Analysis:

For peer review only

Frequencies and percentages were calculated for the language, age rating, alcohol usage and brand appearance. Mean and standard deviation (SD) was calculated for runtime of the film. Proportions of films and intervals were compared for alcohol content between films according to age classification and language using Chi-square test.

Results:

The 30 films included seven (23%) that were U-rated and 23 (77%) UA-rated, and 22 (73%) in Hindi and eight (27%) in regional languages. The regional language included four Tamil, three Telugu and one Malayalam films. The mean (SD) film runtime of the 30 films was 151.2 (12.8) minutes, and their cumulative duration 4535 minutes, which we coded in 923 5-minute intervals. Of these, 229 (25%) were in U and 694 (75%) in UA films, and 665 (72%) in Hindi and 258 (28%) in regional language films. Any alcohol appearance was observed in 195 (21%) 5-minute intervals occurring in 29 (97%) of the films. The proportion of intervals containing alcohol was slightly lower in U-rated (38 intervals, 17%) than UA-rated (157 intervals, 23%) films, though this difference was not statistically significant (p=0.11); and the same in Hindi (140 intervals, 21%) and regional language (55 intervals, 21%) films (p=0.94).

For peer review only - http://bmjopen.bmj.com/site/about/guidelines.xhtml

For peer review only

Actual alcohol use appeared in 25 (83%) films, in 90 (10%) intervals. All seven U-rated films included actual alcohol use, which occurred in 19 (50%) of the 38 intervals including any alcohol imagery, with implied use and other alcohol references occurring in 11 (29%) and 8 (11%) of alcohol intervals respectively. Actual alcohol use occurred in 18 (78%) of the 23 UA rated films in 71 (45%) of the 157 intervals including alcohol, while implied use and other alcohol references occurred in 55 (35%) and 37 (24%) of alcohol intervals respectively. Actual alcohol use occurred in 18 (82%) of the 22 Hindi films, in 64 (46%) of the 140 intervals containing alcohol, with implied use and other alcohol references occurring in 54 (39%) and 25 (17%) of alcohol intervals respectively; and in 7 of the 8 regional language films in 26 (47%) of the 55 intervals containing alcohol, with implied use and other alcohol references each occurring in 12 (22%) and 20 (40%) alcohol intervals respectively as shown in Table 1.

Alcohol brand appearances occurred in ten intervals in five films, one of which was U-rated and one in a regional language. The brands, Signature, Royalstag, Carlsberg, Sula Wine, Old Smuggler, Kingfisher Premium, Kisset, Mcdonald, Brown Horse and Tuborg appeared once each, and Vat 69 twice.

Discussion:

For peer review only - http://bmjopen.bmj.com/site/about/guidelines.xhtml

For peer review only

This study, to our knowledge the first of its kind in India, demonstrates that alcohol imagery occurred in almost all of this sample of films popular in India, all of which were classified by the CBFC in India as suitable either for unrestricted viewing by children (U), or for by children under the age of 12 with parental guidance (UA).15 Most of the alcohol imagery comprised actual or implied use. In contrast with our earlier analysis of tobacco imagery in these films[13], we found no evidence of greater alcohol content in films made in local languages. However, the proportion of both U and UA-rated films containing alcohol was similarly high and although the proportion of intervals containing alcohol was slightly lower in U-rated than in UA-rated films, this difference was not statistically significant. These findings suggest that films are a significant source of exposure to alcohol imagery for children and young people in India, and that the CBFC film classification system, which offers guidance on glorification or justification of drinking alcohol[15], is not currently exerting appreciable influence on overall alcohol content.

Coding films is time-consuming so we confined our study to the most popular films by selecting the top ten box office hit national films in each of three years. These did not include any A- or S-rated films, so our findings related entirely to films classified by the

For peer review only - http://bmjopen.bmj.com/site/about/guidelines.xhtml

CBFC as suitable for young people. We used a 5-minute interval coding method used previously in film content analysis[14] to provide semi-quantitative estimates of alcohol content. Our findings are consistent with reports from other countries: Hanewinkel et al, in a study in 2007 [16], reported alcohol imagery in 88% of 398 films coded, similar to another six-country study 10 published in 2014, wherein 86% of 655 films had alcohol content. The low occurrence of branding in the present study contrasts however with earlier work in the UK, which found frequent occurrences of American beer brands in popular UK films[11].

Exposure of young people to alcohol imagery is unlikely to be limited to that in films watched in cinemas, because films are only one of many entertainment media through which children are exposed to alcohol imagery, and children consume a growing range of media, including YouTube, Facebook, Instagram, Netflix and Amazon Prime, that have been shown to contain alcohol imagery[17-19.] However, films represent an important source of exposure, not only in terms of the cinema audiences they generate but also for the potentially wider audiences reached when films are shown on television. According to a Broadcast Audience Research Council (BARC) report,

For peer review only - http://bmjopen.bmj.com/site/about/guidelines.xhtml

young people represent 33% of total television viewership and films comprise more than half of that viewing in India [20].

Film classification bodies such as the CBFC (and, for example, the British Board of Film Classification) tend to consider alcohol imagery as relevant to age classification only if it involves glamorisation or glorification, but evidence on the effect of tobacco exposure in films suggests that these considerations are unimportant: all tobacco imagery promotes tobacco use, irrespective of who, how, or in what other circumstances the product is consumed [21]. To date, evidence on exposure to alcohol imagery is less developed and further work is needed to establish the extent of this effect. However, the prudent approach to avoidable risks is to avoid them, and since the inclusion of alcohol imagery in films aimed at children is entirely avoidable, protecting children from future alcohol use and consequent problems justifies more rigorous controls on the alcohol content of films aimed at children and young people in India.

Acknowledgments: The authors appreciate the assistance of the project staff of the Preventing smoking uptake study in procuring and coding films.

For peer review only - http://bmjopen.bmj.com/site/about/guidelines.xhtml

References:

1. Gowing LR, Ali RL, Allsop S, Marsden J, Turf EE, West R, et al. Global statistics on addictive behaviours: 2014 status report. *Addiction*. 2015;110(6):904–19.

2. WHO. Global status report on alcohol and health. World Health Organization. 2018. Available from: http://www.who.int/substance_abuse/publications/global_alcohol_report/msbgsruprofiles.pdf

3. Griswold MG, Fullman N, Hawley C, Arian N, Zimsen SRM, Tymeson HD, et al. Alcohol use and burden for 195 countries and territories, 1990-2016: A systematic analysis for the Global Burden of Disease Study 2016. *Lancet*. 2018;392(10152):1015–35. Available from: http://dx.doi.org/10.1016/S0140-6736(18)31310-2

4. Manthey J, Shield KD, Rylett M, Hasan OSM, Probst C, Rehm J. Global alcohol exposure between 1990 and 2017 and forecasts until 2030: a modelling study. *Lancet*. 2019;393(10190):2493–502.

For peer review only - http://bmjopen.bmj.com/site/about/guidelines.xhtml

5. Solanki MS. Alcohol Use Disorder. National Health Portal. 2015. Available from: https://www.nhp.gov.in/healthlyliving/alcohol-use-disorder

6. NFHS 4. National Family Health Survey. Mumbai; 2016. Available from: http://rchiips.org/nfhs/factsheet_NFHS-4.shtml

7. Soundararajan S, Narayanan G, Agrawal A, Prabhakaran D. Relation between age at first alcohol drink & adult life drinking patterns in alcohol-dependent patients. *Indian J Med Res*. 2019;146(5):606–11.

8. Johnson PR, Banu S, Ashok M V. Severity of alcoholism in Indian males: Correlation with age of onset and family history of alcoholism. *Indian J Psychiatry*. 2019;52(3):243–9.

9. Ambekar A, Chadda RK, Khandelwal SK, Rao R, Mishra AK, Agrawal A. Magnitude of Substance Use in India. Ministry of Social Justice and Empowerment Government of India. 2019. Available from: http://socialjustice.nic.in/writereaddata/UploadFile/Magnitude_Substance_Use_India_REPORT.pdf

For peer review only - http://bmjopen.bmj.com/site/about/guidelines.xhtml

10. Hanewinkel R, Sargent JD, Hunt K, Sweeting H, Engels RCME, Scholte RHJ, et al. Portrayal of alcohol consumption in movies and drinking initiation in low-risk adolescents. *Pediatrics*. 2014;133(6):973–82.

11. Lyons A, Mcneill A, Gilmore I, Britton J. Alcohol imagery and branding, and age classification of films popular in the UK. *Int J Epidemiol*. 2011;40(5):1411–9.

12. Box Office India. [cited 2019 Jan 20]. Available from: https://www.boxofficeindia.com/

13. Kulkarni MM, Kamath VG, Cranwell J, Britton J, Nazar GP, Arora M, et al. Assessment of tobacco imagery and compliance with tobacco-free rules in popular Indian films. *Tob Control*. 2019;1–3.

14. Lyons A, Mcneill A, Britton J. Alcohol imagery on popularly viewed television in the UK. *J Public Health*. 2013;36(3):426–34.

15. Ministry of Information and Broadcasting. Central Board of Film Certification. Available from: https://www.cbfcindia.gov.in/main/guidelines.html

16. Hanewinkel R, Tanski SE, Sargent JD. Exposure to alcohol use in motion pictures and teen drinking in Germany. *Int J Epidemiol*. 2007;36:1068–77.

For peer review only - http://bmjopen.bmj.com/site/about/guidelines.xhtml

For peer review only

17. Cranwell J, Murray R, Lewis S, Dockrell M, Britton J. Adolescents' exposure to tobacco and alcohol content in YouTube music videos . *Addiction*. 2015;110(4):703–11.

18. Hendriks H, Van den Putte B, Gebhardt WA, Moreno MA. Social Drinking on Social Media: Content Analysis of the Social Aspects of Alcohol-Related Posts on Facebook and Instagram. *J Med Internet Res*. 2018;20(6):e226.

19. Barker AB, Smith J, Hunter A, et al. Quantifying tobacco and alcohol imagery in Netflix and Amazon Prime instant video original programming accessed from the UK: a content analysis. *BMJ Open.* 2019;9:e025807.

20. What Young India Watches. BARC India Newsletter. 2018 Nov 7;(2). Available from: https://www.barcindia.co.in/resources/What Young India Watches.pdf

21. National Center for Chronic Disease Prevention and Health Promotion (US) Office on Smoking and Health. Preventing Tobacco Use Among Youth and Young Adults: A Report of the Surgeon General [Internet]. Centers for Disease Control and Prevention (US). 2012. Available from: https://www.ncbi.nlm.nih.gov/books/NBK99237/

For peer review only

Table 1: Alcohol imagery based on language and CBFC Rating of the films

Type of Alcohol Imagery	Language								Age Rating								All films			
	**Hindi**				**Regional**				**U Rated**				**UA Rated**				**Films**		**Intervals**	
	22 Films		665 Intervals		8 Films		258 Intervals		7 Films		229 Intervals		23 Films		694 Intervals		30		923	
	n	%	n	%	n	%	N	%	N	%	n	%	n	%	n	%	n	%	n	%
Any Alcohol	21	95.5	140	21.0	8	100.0	55	21.3	7	100.0	38	16.6	22	95.7	157	22.6	29	96.6	195	21.1
Actual use	18	81.8	64	9.6	7	87.5	26	10.1	7	100.0	19	8.3	18	78.3	71	10.2	25	83.3	90	9.8
Implied use	18	81.8	54	8.1	5	62.5	12	4.7	5	71.4	11	4.8	18	78.3	55	7.9	23	76.7	66	7.2

For peer review only - http://bmjopen.bmj.com/site/about/guidelines.xhtml

Other references	10	45.5	25	3.8	5	62.5	20	7.8	4	57.1	8	3.5	12	52.2	37	5.3	15	50.0	45	4.9
Alcohol branding	4	18.2	7	1.1	1	12.5	3	0.5	1	14.3	1	0.4	4	17.4	9	1.3	5	16.7	10	1.1

For peer review only - http://bmjopen.bmj.com/site/about/guidelines.xhtml

Characteristics of the Coded Films for Alcohol Imagery

Film Name	Language	Genre	Age Rating	Total number of 5 minutes intervals	Number and Percentage of Alcohol Imagery
Ae Dil Hai Mushkil	Hindi	Drama, Musical Music	UA	31	24(77.4)
Raees	Hindi	Action, Crime, Drama	UA	27	17(63.0)
Kabali	Tamil	Drama, Crime	UA	29	16(55.2)
Tanu Weds Manu	Hindi	Drama, Comedy, Romance	UA	25	13(52.0)
Rustom	Hindi	Crime, Drama, Thriller	UA	29	13(44.8)
Welcome Back	Hindi	Drama, Crime	UA	29	10(34.5)
Pulimurugan	Malyalam	Action	U	33	10(30.3)
Juduwaa 2	Hindi	Action, Comedy	UA	30	9(30.0)
Shrimanthudu	Telugu	Drama, Action	UA	32	9(28.1)
Airlift	Hindi	Drama, History	UA	25	6(24.0)
Kabil	Hindi	Drama	UA	27	6(22.2)
Prem Ratan Dan Payo	Hindi	Action, Drama, Musical	U	32	6(18.8)
Mersal	Tamil	Action, Thriller	U	34	6(17.6)
Shivaay	Hindi	Action, Adventure, Drama	UA	30	5(16.7)
Bahubali	Telugu	Action, Drama, Fantasy	UA	31	5(16.1)
Dilwale	Hindi	Action, Comedy	UA	32	5(15.6)
Dangal	Hindi	Action, Biography, Drama	U	33	5(15.2)
Tubelight	Hindi	Drama, History	U	27	4(14.8)
Baby	Hindi	Action, Thriller	UA	32	4(12.5)
Sulthan	Hindi	Drama, Sport	UA	33	4(12.1)
I	Tamil	Romantic Thriller	U	34	4(11.8)
Their	Tamil	Action	UA	31	3(9.7)
M S Dhoni	Hindi	Biography, Drama, Sport	U	36	3(8.3)
Bajirao Mastani	Hindi	Drama, Comedy, Action	UA	32	2(6.3)
Bahubali 2	Telugu	Action, Drama, Fantasy	UA	34	2(5.9)
Toilet Ek Pream Katha	Hindi	Comedy, Drama	UA	30	1(3.3)
Goolmaal Again	Hindi	Comedy	UA	31	1(3.2)
Bhajarangi Bhaijan	Hindi	Drama, Comedy, Action	UA	31	1(3.2)
Tiger Zinda Hai	Hindi	Action, Thriller	UA	33	1(3.0)
Secret Super Star	Hindi	Drama	UA	30	0(0.0)

For peer review only

Quantifying Audio Visual Alcohol Imagery in Popular Indian Films: A Content Analysis

Journal:	*BMJ Open*
Manuscript ID	bmjopen-2020-040630.R1
Article Type:	Original research
Date Submitted by the Author:	04-Jan-2021
Complete List of Authors:	Kulkarni, Muralidhar; Kasturba Medical College Manipal, Community Medicie<br>Britton, John; University of Nottingham, Division of Epidemiology and Public Health<br>Cranwell, Jo; University of Bath, Department for Health<br>Arora, Monika; HRIDAY; Public Health Foundation of India, Health Promotion Division<br>Nazar, Gaurang; HRIDAY; Public Health Foundation of India, Health Promotion Division<br>Mullapudi, Somya; Kasturba Medical College Manipal, Community Medicine<br>Kamath, Veena; Kasturba Medical College Manipal, Community Medicine
**Primary Subject Heading**:	Epidemiology
Secondary Subject Heading:	Epidemiology, Public health, Health policy
Keywords:	Health policy < HEALTH SERVICES ADMINISTRATION & MANAGEMENT, EPIDEMIOLOGY, PUBLIC HEALTH, Child & adolescent psychiatry < PSYCHIATRY, Community child health < PAEDIATRICS

I, the Submitting Author has the right to grant and does grant on behalf of all authors of the Work (as defined in the below author licence), an exclusive licence and/or a non-exclusive licence for contributions from authors who are: i) UK Crown employees; ii) where BMJ has agreed a CC-BY licence shall apply, and/or iii) in accordance with the terms applicable for US Federal Government officers or employees acting as part of their official duties; on a worldwide, perpetual, irrevocable, royalty-free basis to BMJ Publishing Group Ltd ("BMJ") its licensees and where the relevant Journal is co-owned by BMJ to the co-owners of the Journal, to publish the Work in this journal and any other BMJ products and to exploit all rights, as set out in our licence.

The Submitting Author accepts and understands that any supply made under these terms is made by BMJ to the Submitting Author unless you are acting as an employee on behalf of your employer or a postgraduate student of an affiliated institution which is paying any applicable article publishing charge ("APC") for Open Access articles. Where the Submitting Author wishes to make the Work available on an Open Access basis (and intends to pay the relevant APC), the terms of reuse of such Open Access shall be governed by a Creative Commons licence – details of these licences and which Creative Commons licence will apply to this Work are set out in our licence referred to above.

Other than as permitted in any relevant BMJ Author's Self Archiving Policies, I confirm this Work has not been accepted for publication elsewhere, is not being considered for publication elsewhere and does not duplicate material already published. I confirm all authors consent to publication of this Work and authorise the granting of this licence.

For peer review only

Quantifying Audio Visual Alcohol Imagery in Popular Indian Films: A Content Analysis

Rohith Bhagawath[1], Muralidhar Kulkarni[1], John Britton[2*], Jo Cranwell[3], Monika Arora[4,5], Gaurang P Nazar[4,5], Somya Mullapudi[1], Veena G Kamath[1].

Correspondence to:

Professor John Britton

UK Center for Tobacco and Alcohol Studies, Division of Epidemiology and Public Health,

University of Nottingham

E-mail: j.britton@outlook.com

[1] Department of Community Medicine, Kasturba Medical College, MAHE, Manipal, India

For peer review only

[2] Division of Epidemiology and Public Health, University of Nottingham

[3] Tobacco Control Research Group, Department for Health, University of Bath, Bath, UK

[4] HRIDAY, Delhi, India

[5] Health Promotion Division, Public Health Foundation of India, Delhi, India

For peer review only - http://bmjopen.bmj.com/site/about/guidelines.xhtml

Word Count: 1954

Key words: Alcohol, Coding, Films, Adolescents, Media, Imagery.

For peer review only

For peer review only - http://bmjopen.bmj.com/site/about/guidelines.xhtml

Abstract

Objectives: Though exposure to alcohol imagery in films is a significant determinant of uptake and severity of alcohol consumption among young people, there is poor evidence regarding the content of alcohol imagery in films in low and middle-income countries. We have measured alcohol imagery content and branding in popular Indian films, in total and in relation to language and age rating.

Design: In this observational study we measured alcohol imagery semi-quantitatively using 5-minute interval coding. We coded each interval according to whether it contained alcohol imagery or brand appearances.

Setting: India.

Participants: None. Content analysis of a total of 30 National Box office hit films over a period of three years from 2015-17.

Primary and secondary outcome measures: To assess alcohol imagery in Indian films and its distribution in relation to age and language rating has been determined.

Results: The 30 films included 22 (73%) Hindi films and eight (27%) in regional languages. Seven (23%) were rated suitable for viewing by all ages (U), and 23 (77%)

For peer review only

rated as suitable for viewing by children subject to parental guidance for those aged under 12 (UA). Any alcohol imagery was seen in 97% of the films, with 195 of a total of 923 5-minute intervals, and actual alcohol use in 25 (83%) films, in 90 (10%) intervals. The occurrence of these and other categories of alcohol imagery was similar in U and UA-rated films, and in Hindi and local language films. Episodes of alcohol branding occurred in ten intervals in five films.

Conclusion: Almost all films popular in India contain alcohol imagery, irrespective of age rating and language. Measures need to be undertaken to limit alcohol imagery in Indian films to protect the health of young people, and to monitor alcohol imagery in other social media platforms in future.

For peer review only

Strengths and limitations of this study:

- Our study, to the best of our knowledge is the first of its kind to measure alcohol imagery in popular films in India.
- The study demonstrates that almost all popular Indian films contain alcohol imagery.
- We included only top ten national box office hit films in the years 2015, 2016 and 2017 since coding films is time-consuming.

For peer review only - http://bmjopen.bmj.com/site/about/guidelines.xhtml

- Films in only four Indian languages figured in the box office hits; while films in several other regional languages did not.
- Although our study did not include any A (Adult) or S-rated (Restricted to specialised audience) films, our findings related entirely to films classified by the CBFC as suitable for young people.

For peer review only - http://bmjopen.bmj.com/site/about/guidelines.xhtml

Background:

Consumption of alcohol is a major avoidable cause of morbidity and mortality [1]. According to the WHO, there are 2.3 billion current alcohol users around the world [2], and in 2016 alcohol consumption led to 2.8 million deaths [3]. Global alcohol consumption has increased by 38% in the last decade [4], indicating that the burden of morbidity and mortality caused by alcohol is likely to grow.

Alcohol consumption is a significant public health problem in India [5]. Although rare among women, nearly one in three men now consume alcohol [6], with an average intake of 18.3 litres per year[2]. Although the mean age at which people begin consuming alcohol is 21 years[7,8], 1.3% of children aged 10-17 report alcohol consumption [9], and studies reveal that early onset of use of alcohol in India correlates with chronic heavy drinking patterns later in adult life[7,8].

Research among adolescents in developed countries indicates that in addition to paid-for advertising, exposure to media imagery of alcohol products and alcohol consumption, particularly in films, plays an important role in promoting the uptake of alcohol consumption among young people[10]. However, while national film age-rating or classification systems typically include restrictions on alcohol imagery allowed in

For peer review only - http://bmjopen.bmj.com/site/about/guidelines.xhtml

films rated suitable for viewing by children or young people, analysis of alcohol imagery in popular UK films demonstrates that alcohol content continues to occur frequently in films marketed to these groups[11]. To our knowledge, however there here has to date been no evaluation of the extent to which popular films in India include alcohol imagery. This study was therefore carried out to quantify alcohol imagery, in relation to the age classification and language of the film, in a sample of the most popular films in India in the years 2015-2017.

Methods:

After obtaining Institutional ethics approval (IEC: 893/2018), we used Indian national box office ratings data to identify the top 10 Indian films by box office revenue in each of the years 2015, 2016 and 2017 [12]. The CBFC (Central Board of Film Certification) age rating (comprising U (Unrestricted Public Exhibition), UA (Parental Guidance for children below the age of 12 years), A (Restricted to adults) and S (Restricted to any special class of persons)) along with the genre and language of each film was noted. We used the validated 5-minute interval coding,[13,14] as described in previous studies, to code the presence of alcohol imagery in each interval as any use, actual use,

For peer review only

implied use, other alcohol references and alcohol brand appearances. Actual use of alcohol was coded if an actor/actress in the film was shown consuming alcohol, while implied alcohol use typically involved verbal comments related to alcohol or non-verbal actions such as holding a glass or a bottle appearing to contain an alcoholic drink. Other alcohol references usually comprised the appearance of alcohol bottles or beer mugs. An occurrence in any of the above categories was coded as present or absent for each interval. Multiple appearances in the same category in the same interval were coded as a single event, and appearances in different categories as separate events. The same appearance transitioning into two or more intervals was coded as two or more events, as appropriate. Accuracy of results was ensured by two coders independently coding each of the movies with quality check done by two other coders. Any discrepancy was cleared after discussing with the investigators of the study. For alcohol branding we noted occurrences of clear, unambiguous alcohol branding on-screen, sub-grouped as branding on a product used in a scene, branding on a product not used in a scene, branded merchandise, advertisements visible in scenes of alcohol content and any other alcohol-related advertisement. The total number of alcohol brands shown in were counted and listed.

For peer review only - http://bmjopen.bmj.com/site/about/guidelines.xhtml

Patient and public involvement: No patients were involved in this study.

Analysis:

Frequencies and percentages were calculated for the language, age rating, alcohol usage and brand appearance. Mean and standard deviation (SD) was calculated for runtime of the film. Proportions of films and intervals were compared for alcohol content between films according to age classification and language using Chi-square test.

Results:

The 30 films included seven (23%) that were U-rated and 23 (77%) UA-rated, and 22 (73%) in Hindi and eight (27%) in regional languages. The regional language included four Tamil, three Telugu and one Malayalam films. The mean (SD) film runtime of the 30 films was 151.2 (12.8) minutes, and their cumulative duration 4535 minutes, which we coded in 923 5-minute intervals. Of these, 229 (25%) were in U and 694 (75%) in UA films, and 665 (72%) in Hindi and 258 (28%) in regional language films. The list of the films with their language, genre, age rating and the number of intervals is provided

For peer review only

For peer review only - http://bmjopen.bmj.com/site/about/guidelines.xhtml

in the supplementary file. Any alcohol appearance was observed in 195 (21%) 5-minute intervals occurring in 29 (97%) of the films. The proportion of intervals containing alcohol was slightly lower in U-rated (38 intervals, 17%) than UA-rated (157 intervals, 23%) films, though this difference was statistically not significant ($p>0.05$); and the same in Hindi (140 intervals, 21%) and regional language (55 intervals, 21%) films ($p>0.05$).

Actual alcohol use appeared in 25 (83%) films, in 90 (10%) intervals. All seven U-rated films included actual alcohol use, which occurred in 19 (50%) of the 38 intervals including any alcohol imagery, with implied use and other alcohol references occurring in 11 (29%) and 8 (11%) of alcohol intervals respectively. Actual alcohol use occurred in 18 (78%) of the 23 UA rated films in 71 (45%) of the 157 intervals including alcohol, while implied use and other alcohol references occurred in 55 (35%) and 37 (24%) of alcohol intervals respectively. Actual alcohol use occurred in 18 (82%) of the 22 Hindi films, in 64 (46%) of the 140 intervals containing alcohol, with implied use and other alcohol references occurring in 54 (39%) and 25 (17%) of alcohol intervals respectively; and in 7 of the 8 regional language films in 26 (47%) of the 55 intervals

For peer review only - http://bmjopen.bmj.com/site/about/guidelines.xhtml

containing alcohol, with implied use and other alcohol references each occurring in 12 (22%) and 20 (40%) alcohol intervals respectively as shown in Table 1.

Alcohol brand appearances occurred in ten intervals in five films, one of which was U-rated and one in a regional language. The brands, Signature, Royalstag, Carlsberg, Sula Wine, Old Smuggler, Kingfisher Premium, Kisset, Mcdonald, Brown Horse and Tuborg appeared once each, and Vat 69 twice.

Discussion:

This study, to our knowledge the first of its kind in India, demonstrates that alcohol imagery occurred in almost all of this sample of films popular in India, all of which were classified by the CBFC in India as suitable either for unrestricted viewing by children (U), or for by children under the age of 12 with parental guidance (UA).[15] Most of the alcohol imagery comprised actual or implied use. In contrast with our earlier analysis of tobacco imagery in these films[13], we found no evidence of greater alcohol content in films made in local languages. However, the proportion of both U and UA-rated films containing alcohol was similarly high and although the proportion of intervals containing alcohol was slightly lower in U-rated than in UA-rated films, this difference was not statistically significant. These findings suggest that films are a significant

For peer review only - http://bmjopen.bmj.com/site/about/guidelines.xhtml

source of exposure to alcohol imagery for children and young people in India, and that the CBFC film classification system, which offers guidance on glorification or justification of drinking alcohol[15], is not currently exerting appreciable influence on overall alcohol content.

Coding films is time-consuming so we confined our study to the most popular films by selecting the top ten box office hit national films in each of three years. These did not include any A- or S-rated films, so our findings related entirely to films classified by the CBFC as suitable for young people. Films in India are made in 35 languages[16] while we coded only top 30 films that comprised of films in four languages. There is a need to code films in other languages to monitor alcohol imagery and take appropriate measures.

A limitation in the methodology that we used 5 minute intervals as coding units and thereby multiple scenes, if present in a single interval might be underrated.[11] Coding can be done by methods such as 5 min interval coding[17], 1 min interval coding[18], using scene breaks to define intervals[19,20] or methods of continuous measurements[21,22], with each having strengths and weaknesses. However, the 5 min interval method has proved to be a sensitive means of capturing behavioural variation[23] and an effective

For peer review only - http://bmjopen.bmj.com/site/about/guidelines.xhtml

For peer review only

semi-quantitative method of measurement which balances accuracy with the logistic need to conduct highly time-consuming measurements efficiently. This method has been used in many studies and has been used in the present study also.[24,25] to provide semi-quantitative estimates of alcohol content. Our findings are consistent with reports from other countries: Hanewinkel et al, in a study in 2007 [26], reported alcohol imagery in 88% of 398 films coded, similar to another six-country study 10 published in 2014, wherein 86% of 655 films had alcohol content. The low occurrence of branding in the present study contrasts however with earlier work in the UK, which found frequent occurrences of American beer brands in popular UK films[11].

Exposure of young people to alcohol imagery is unlikely to be limited to that in films watched in cinemas, because films are only one of many entertainment media through which children are exposed to alcohol imagery, and children consume a growing range of media, including social media, that have been shown to contain alcohol imagery[27-29.] However, films represent an important source of exposure, not only in terms of the cinema audiences they generate but also for the potentially wider audiences reached when films are shown on television. According to a Broadcast Audience Research

For peer review only

Council (BARC) report, young people represent 33% of total television viewership and films comprise more than half of that viewing in India [30].

Film classification bodies such as the CBFC (and, for example, the British Board of Film Classification) tend to consider alcohol imagery as relevant to age classification only if it involves glamorisation or glorification, but evidence on the effect of tobacco exposure in films suggests that these considerations are unimportant: all tobacco imagery promotes tobacco use, irrespective of who, how, or in what other circumstances the product is consumed [31]. To date, evidence on exposure to alcohol imagery is less developed and further work is needed to establish the extent of this effect. However, the prudent approach to avoidable risks is to avoid them, and since the inclusion of alcohol imagery in films aimed at children is entirely avoidable, protecting children from future alcohol use and consequent problems justifies more rigorous controls on the alcohol content of films aimed at children and young people in India.

Acknowledgments: The authors appreciate the assistance of the project staff of the Preventing smoking uptake study in procuring and coding films.

For peer review only - http://bmjopen.bmj.com/site/about/guidelines.xhtml

Contributorship statement: JB, MMK and RB conceptualised the study. The coding sheet was developed by RB, MMK, JC and GPN. Coding analysis was done by RB, MMK and VGK. Interpretation of data and drafting the manuscript was done by RB, MMK, VGK, SM and MA. Final approval of the version was read and approved by all authors.

Competing Interests: None to declare.

Funding Statement:

This work was supported by the Medical Research Council [grant number MR/P008933/1] of the United Kingdom under the Global Alliance for Chronic Lung diseases program.

Data Sharing Statement: Additional data on the alcohol content found in each film are available on request from rohit.bhagawath@manipal.edu.

For peer review only

For peer review only - http://bmjopen.bmj.com/site/about/guidelines.xhtml

References:

1. Gowing LR, Ali RL, Allsop S, Marsden J, Turf EE, West R, et al. Global statistics on addictive behaviours: 2014 status report. *Addiction*. 2015;110(6):904–19.

2. WHO. Global status report on alcohol and health. World Health Organization. 2018. Available from: http://www.who.int/substance_abuse/publications/global_alcohol_report/msbgsruprofiles.pdf

3. Griswold MG, Fullman N, Hawley C, Arian N, Zimsen SRM, Tymeson HD, et al. Alcohol use and burden for 195 countries and territories, 1990-2016: A systematic analysis for the Global Burden of Disease Study 2016. *Lancet*. 2018;392(10152):1015–35. Available from: http://dx.doi.org/10.1016/S0140-6736(18)31310-2

4. Manthey J, Shield KD, Rylett M, Hasan OSM, Probst C, Rehm J. Global alcohol exposure between 1990 and 2017 and forecasts until 2030: a modelling study. *Lancet*. 2019;393(10190):2493–502.

For peer review only - http://bmjopen.bmj.com/site/about/guidelines.xhtml

For peer review only

5. Solanki MS. Alcohol Use Disorder. National Health Portal. 2015. Available from: https://www.nhp.gov.in/healthlyliving/alcohol-use-disorder

6. NFHS 4. National Family Health Survey. Mumbai; 2016. Available from: http://rchiips.org/nfhs/factsheet_NFHS-4.shtml

7. Soundararajan S, Narayanan G, Agrawal A, Prabhakaran D. Relation between age at first alcohol drink & adult life drinking patterns in alcohol-dependent patients. *Indian J Med Res*. 2019;146(5):606–11.

8. Johnson PR, Banu S, Ashok M V. Severity of alcoholism in Indian males: Correlation with age of onset and family history of alcoholism. *Indian J Psychiatry*. 2019;52(3):243–9.

9. Ambekar A, Chadda RK, Khandelwal SK, Rao R, Mishra AK, Agrawal A. Magnitude of Substance Use in India. Ministry of Social Justice and Empowerment Government of India. 2019. Available from: http://socialjustice.nic.in/writereaddata/UploadFile/Magnitude_Substance_Use_India_REPORT.pdf

For peer review only - http://bmjopen.bmj.com/site/about/guidelines.xhtml

10. Hanewinkel R, Sargent JD, Hunt K, Sweeting H, Engels RCME, Scholte RHJ, et al. Portrayal of alcohol consumption in movies and drinking initiation in low-risk adolescents. *Pediatrics*. 2014;133(6):973–82.

11. Lyons A, Mcneill A, Gilmore I, Britton J. Alcohol imagery and branding, and age classification of films popular in the UK. *Int J Epidemiol*. 2011;40(5):1411–9.

12. Box Office India. [cited 2019 Jan 20]. Available from: https://www.boxofficeindia.com/

13. Kulkarni MM, Kamath VG, Cranwell J, Britton J, Nazar GP, Arora M, et al. Assessment of tobacco imagery and compliance with tobacco-free rules in popular Indian films. *Tob Control*. 2019;1–3.

14. Lyons A, Mcneill A, Britton J. Alcohol imagery on popularly viewed television in the UK. *J Public Health*. 2013;36(3):426–34.

15. Ministry of Information and Broadcasting. Central Board of Film Certification. Available from: https://www.cbfcindia.gov.in/main/guidelines.html

16. UNESCO Institute for Statistics. (2016). *Record Number of Films Produced.* http://uis.unesco.org/en/news/record-number-films-produced#slideoutmenu

For peer review only - http://bmjopen.bmj.com/site/about/guidelines.xhtml

17. Lyons A, McNeill A, Chen Y, et al. Tobacco and tobacco branding in films most popular in the UK from 1989 to 2008. Thorax 2010;65:417-422.

18. Mekemson C, Glik D, Titus K, et al. Tobacco use in popular movies during the past decade. Tob Control 2004;13:400e2.

19. Dalton MA, Tickle JJ, Sargent JD, et al. The incidence and context of tobacco use in popular movies from 1988 to 1997. Prev Med 2002;34:516e23.

20. Worth KA, Dal Cin S, Sargent JD. Prevalence of smoking among major movie characters 1996e2004. Tob Control 2006;15:442e6.

21. Adachi-Mejia AM, Dalton MA, Gibson JJ, et al. Tobacco brand appearances in movies before and after the master settlement agreement. JAMA 2005;293:2341e2.

22. Sargent JD, Tickle JJ, Beach ML, et al. Brand appearances in contemporary cinema films and contribution to global marketing of cigarettes. Lancet 2001;357:29e32.

23. Harrop A, Daniels M. Methods of time sampling: a reappraisal of momentary time sampling and partial interval recording. J Appl Behav Anal 1986;19:73e7.

For peer review only

For peer review only - http://bmjopen.bmj.com/site/about/guidelines.xhtml

24. Everett SA, Schnuth RL, Tribble JL. Tobacco and alcohol use in top-grossing American films. J Community Health 1998;23:317e24.

25. Escamilla G, Cradock AL, Kawachi I. Women and smoking in Hollywood movies: a content analysis. Am J Public Health 2000;90:412e4.

26. Hanewinkel R, Tanski SE, Sargent JD. Exposure to alcohol use in motion pictures and teen drinking in Germany. *Int J Epidemiol*. 2007;36:1068–77.

27. Cranwell J, Murray R, Lewis S, Dockrell M, Britton J. Adolescents' exposure to tobacco and alcohol content in YouTube music videos . *Addiction*. 2015;110(4):703–11.

28. Hendriks H, Van den Putte B, Gebhardt WA, Moreno MA. Social Drinking on Social Media: Content Analysis of the Social Aspects of Alcohol-Related Posts on Facebook and Instagram*. J Med Internet Res*. 2018;20(6):e226.

29. Barker AB, Smith J, Hunter A, et al. Quantifying tobacco and alcohol imagery in Netflix and Amazon Prime instant video original programming accessed from the UK: a content analysis. *BMJ Open.* 2019;9:e025807.

For peer review only - http://bmjopen.bmj.com/site/about/guidelines.xhtml

30. What Young India Watches. BARC India Newsletter. 2018 Nov 7;(2). Available from: https://www.barcindia.co.in/resources/What Young India Watches.pdf

31. National Center for Chronic Disease Prevention and Health Promotion (US) Office on Smoking and Health. Preventing Tobacco Use Among Youth and Young Adults: A Report of the Surgeon General [Internet]. Centers for Disease Control and Prevention (US). 2012. Available from: https://www.ncbi.nlm.nih.gov/books/NBK99237/

For peer review only

For peer review only - http://bmjopen.bmj.com/site/about/guidelines.xhtml

Table 1: Alcohol imagery based on language and CBFC Rating of the films

Type of Alcohol Imagery	Language								Age Rating								All films			
	**Hindi**				**Regional**				**U Rated**				**UA Rated**				**Films**		**Intervals**	
	22 Films		665 Intervals		8 Films		258 Intervals		7 Films		229 Intervals		23 Films		694 Intervals		30		923	
	n	%	n	%	n	%	N	%	N	%	n	%	n	%	n	%	n	%	n	%
Any Alcohol	21	95.5	140	21.0	8	100.0	55	21.3	7	100.0	38	16.6	22	95.7	157	22.6	29	96.6	195	21.1
Actual use	18	81.8	64	9.6	7	87.5	26	10.1	7	100.0	19	8.3	18	78.3	71	10.2	25	83.3	90	9.8
Implied use	18	81.8	54	8.1	5	62.5	12	4.7	5	71.4	11	4.8	18	78.3	55	7.9	23	76.7	66	7.2

For peer review only - http://bmjopen.bmj.com/site/about/guidelines.xhtml

Other references	10	45.5	25	3.8	5	62.5	20	7.8	4	57.1	8	3.5	12	52.2	37	5.3	15	50.0	45	4.9
Alcohol branding	4	18.2	7	1.1	1	12.5	3	0.5	1	14.3	1	0.4	4	17.4	9	1.3	5	16.7	10	1.1

For peer review only

For peer review only - http://bmjopen.bmj.com/site/about/guidelines.xhtml

Characteristics of the Coded Films for Alcohol Imagery

Film Name	Language	Genre	Age Rating	Total number of 5 minutes intervals	Number and Percentage of Alcohol Imagery
Ae Dil Hai Mushkil	Hindi	Drama, Musical Music	UA	31	24(77.4)
Raees	Hindi	Action, Crime, Drama	UA	27	17(63.0)
Kabali	Tamil	Drama, Crime	UA	29	16(55.2)
Tanu Weds Manu	Hindi	Drama, Comedy, Romance	UA	25	13(52.0)
Rustom	Hindi	Crime, Drama, Thriller	UA	29	13(44.8)
Welcome Back	Hindi	Drama, Crime	UA	29	10(34.5)
Pulimurugan	Malyalam	Action	U	33	10(30.3)
Juduwaa 2	Hindi	Action, Comedy	UA	30	9(30.0)
Shrimanthudu	Telugu	Drama, Action	UA	32	9(28.1)
Airlift	Hindi	Drama, History	UA	25	6(24.0)
Kabil	Hindi	Drama	UA	27	6(22.2)
Prem Ratan Dan Payo	Hindi	Action, Drama, Musical	U	32	6(18.8)
Mersal	Tamil	Action, Thriller	U	34	6(17.6)
Shivaay	Hindi	Action, Adventure, Drama	UA	30	5(16.7)
Bahubali	Telugu	Action, Drama, Fantasy	UA	31	5(16.1)
Dilwale	Hindi	Action, Comedy	UA	32	5(15.6)
Dangal	Hindi	Action, Biography, Drama	U	33	5(15.2)
Tubelight	Hindi	Drama, History	U	27	4(14.8)
Baby	Hindi	Action, Thriller	UA	32	4(12.5)
Sulthan	Hindi	Drama, Sport	UA	33	4(12.1)
I	Tamil	Romantic Thriller	U	34	4(11.8)
Their	Tamil	Action	UA	31	3(9.7)
M S Dhoni	Hindi	Biography, Drama, Sport	U	36	3(8.3)
Bajirao Mastani	Hindi	Drama, Comedy, Action	UA	32	2(6.3)
Bahubali 2	Telugu	Action, Drama, Fantasy	UA	34	2(5.9)
Toilet Ek Pream Katha	Hindi	Comedy, Drama	UA	30	1(3.3)
Goolmaal Again	Hindi	Comedy	UA	31	1(3.2)
Bhajarangi Bhaijan	Hindi	Drama, Comedy, Action	UA	31	1(3.2)
Tiger Zinda Hai	Hindi	Action, Thriller	UA	33	1(3.0)
Secret Super Star	Hindi	Drama	UA	30	0(0.0)

For peer review only
